# Stoichiometric plasticity of microbial communities is similar between litter and soil in a tropical rainforest

**DOI:** 10.1038/s41598-017-12609-8

**Published:** 2017-10-02

**Authors:** Nicolas Fanin, Nathalie Fromin, Sandra Barantal, Stephan Hättenschwiler

**Affiliations:** 1 Interaction Soil Plant Atmosphere (ISPA), UMR 1391, INRA - Bordeaux Sciences Agro, 71 avenue Edouard Bourlaux, 33882 Villenave-d’Ornon cedex, France; 2 Centre of Evolutionary and Functional Ecology (CEFE), UMR 5175, CNRS - Université de Montpellier - Université Paul-Valéry Montpellier - EPHE, 1919 route de Mende, 34293 Montpellier, France; 3 School of Biological Sciences, Royal Holloway, University of London, Egham, United Kingdom; 4 PROMES-CNRS, 7 rue du Four Solaire, F-66120 Odeillo, France

## Abstract

Heterotrophic microorganisms are commonly thought to be stoichiometrically homeostatic but their stoichiometric plasticity has rarely been examined, particularly in terrestrial ecosystems. Using a fertilization experiment in a tropical rainforest, we evaluated how variable substrate stoichiometry may influence the stoichiometry of microbial communities in the leaf litter layer and in the underlying soil. C:N:P ratios of the microbial biomass were higher in the organic litter layer than in the underlying mineral soil. Regardless of higher ratios for litter microbial communities, C, N, and P fertilization effects on microbial stoichiometry were strong in both litter and soil, without any fundamental difference in plasticity between these two communities. Overall, N:P ratios were more constrained than C:nutrient ratios for both litter and soil microbial communities, suggesting that stoichiometric plasticity arises because of a decoupling between C and nutrients. Contrary to the simplifying premise of strict homeostasis in microbial decomposers, we conclude that both litter and soil communities can adapt their C:N:P stoichiometry in response to the stoichiometric imbalance of available resources.

## Introduction

The decomposition of organic material is commonly considered as a continuous process from freshly produced – mostly plant-derived – litter to soil organic matter (SOM), with increasing turnover times the older the material gets. However, the traditional view that there is not much difference in the composition and the nutritional requirements of microbial communities and their general functioning along the litter-soil continuum^1^ might have to be revisited according to recent findings. For instance, element-use efficiency, trophic interactions or microbial-driven processes have been shown to vary between litter and soil communities^2–4^. The discrepancies in the response of litter and soil microbial communities to variable resource stoichiometry may be related to contrasting C and nutrient limitations reflecting the distinct available substrates^5^. Indeed, freshly fallen leaf litter is relatively rich in easily decomposable C forms compared to the energy-depleted SOM^6^, but presents considerably wider C:N:P ratios (3007:45:1^7^) than that of the SOM (186:13:1^8^). Stoichiometric plasticity is an important mechanism by which biological communities can respond to the stoichiometric imbalance with their growth substrates. It can arise by either changes in biomass stoichiometry at the organismal level and/or by shifts in community structure^9^. Yet, despite experimental fertilization was shown to influence the C:N:P ratios of plant tissues^10^, the question of how substrate stoichiometry and its alteration affect the stoichiometry of microbial communities along the litter-soil continuum remains poorly studied.

In this study, we used a fully factorial C, N and P fertilization experiment in a tropical rainforest to compare the stoichiometric plasticity of microbial communities in the litter layer to that in the underlying soil. Because litter microbial communities grow on substrates with greater stoichiometric imbalance compared to those in the soil^5^, we hypothesized that microbial communities present wider biomass C:N:P ratios in the litter layer than in the soil (H_1_). Based on the greater range and variability of C:N:P ratios of leaf litter compared to SOM^11^, we hypothesized that litter communities are stoichiometrically more plastic (*i.e*. display a wider range of C:N:P ratios) than in the soil (H_2_). By assessing how substrate stoichiometry and its alteration through fertilization affect the C:N:P stoichiometry of microbes, this study aims to improve our understanding of the stoichiometric plasticity of microbial communities in terrestrial ecosystems.

## Results

### Fertilization effects on substrate C:N:P stoichiometry

**Figure 1:**
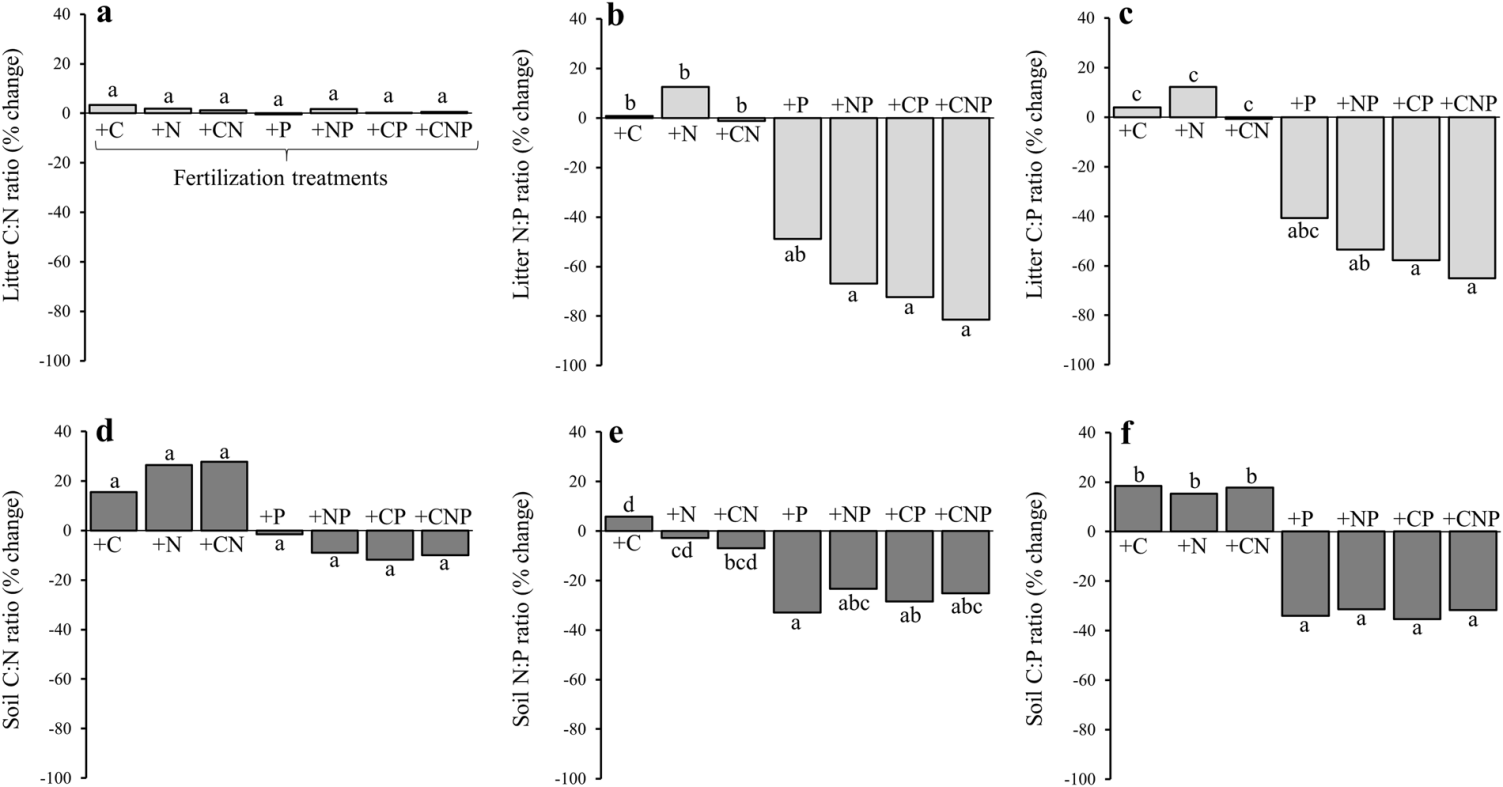
Fertilization effects (+C/+N/+P/+CN/+CP/+NP/+CNP) on (**a**) litter C:N, (**b**) litter N:P and (**c**) litter C:P ratios (light grey) and (**d**) soil C:N, (**e**) soil N:P and (**f**) soil C:P ratios (dark grey). Fertilization effects (% change) were calculated for each individual plot before and after fertilization events (*n* = 5 per treatment). These effects were corrected by accounting for inter-annual variation determined in control plots. Different letters indicate significant differences among treatments.

The fertilization with C and N had no significant effects on the concentrations of C, N, and P and their ratios in either the litter layer or the underlying soil (Tables S1 and S2). In contrast, P fertilization increased P concentrations in both, the litter layer and the underlying soil (Table S1). Higher P concentrations resulted in lower litter N:P (on average −58%) and litter C:P ratios (−60%), as well as lower soil N:P (−28%) and soil C:P ratios (−45%), with no effect on litter or soil C:N ratios (Fig. 1, Tables S1 and S2). Substrate stoichiometry varied from 582:19:1 to 1943:60:1 in the litter compared to 109:8:1 to 229:14:1 in the soil. The observed changes in substrate stoichiometry in response to fertilization were independent of and much larger than pre-treatment differences among our experimental plots (Fig. 1).

### Microbial biomass C:N:P and stoichiometry in litter and soil

**Figure 2:**
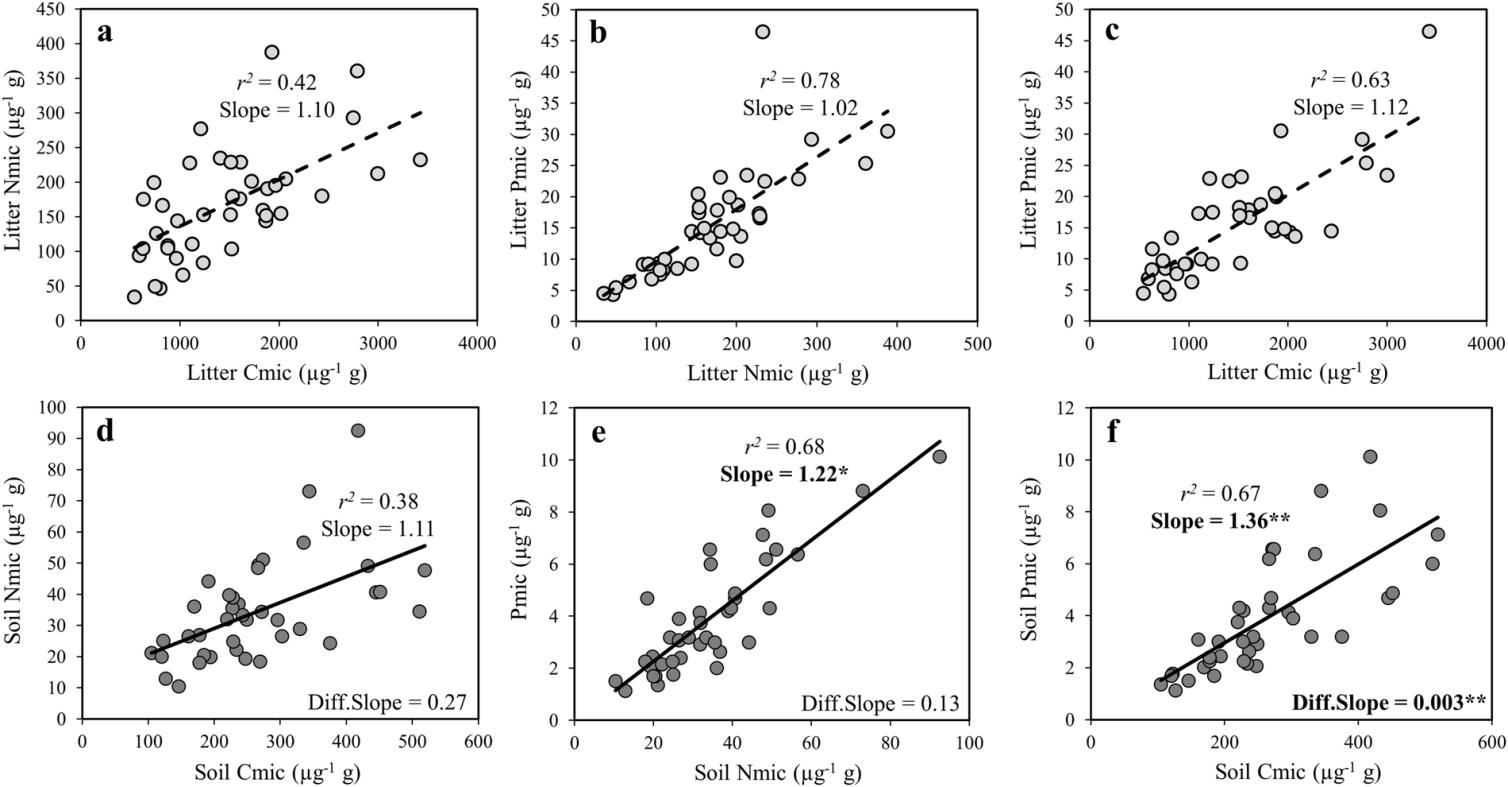
Correlations among Cmic, Nmic and Pmic for litter communities ((**a**–**c**) light grey circles, dashed line, *n* = 40) and soil communities ((**d**–**f**) dark grey circles, full line, *n* = 39). Slopes significantly different from 1 are displayed in boldface: *P < 0.05, **P < 0.01. Diff.slopes indicate differences between slopes when comparing litter to soil (**a**–**d**, **b**–**e**, **c**–**f**).

**Figure 3:**
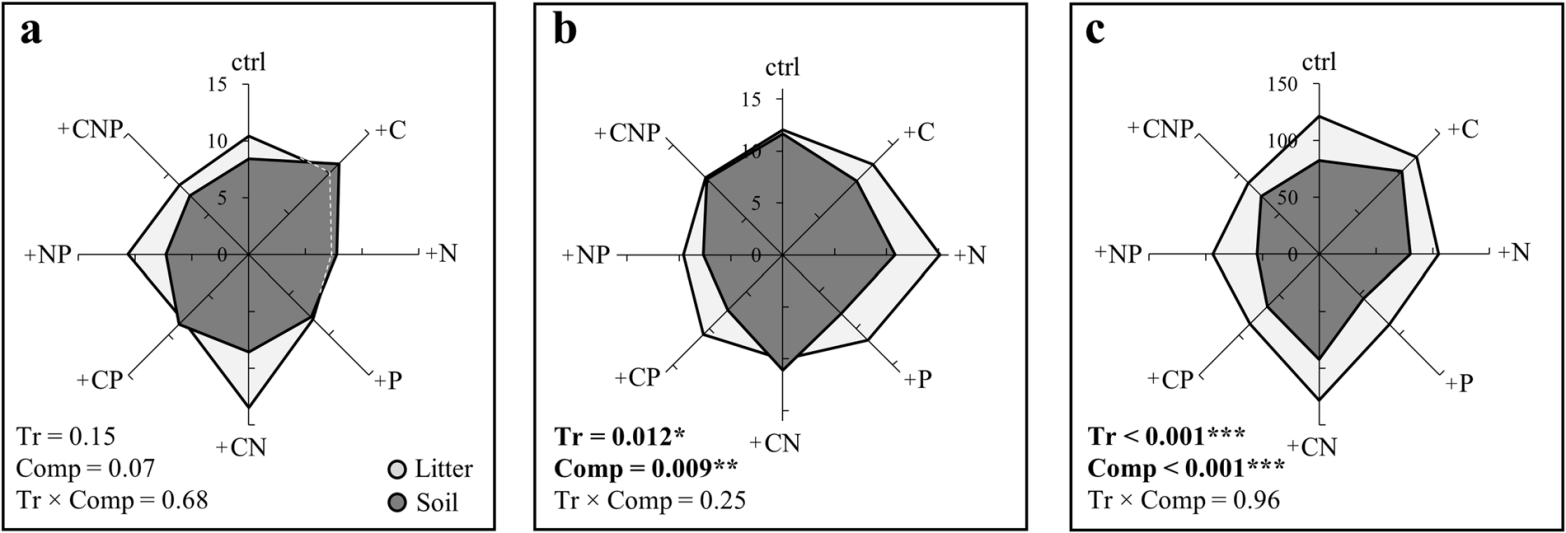
Spider diagrams representing the effects of fertilization treatments on C:Nmic (**a**), N:Pmic (**b**) and C:Pmic (**c**) for litter (light grey, *n* = 40) and soil communities (dark grey, *n* = 39). Tr = treatment (ctrl/+C/+N/+P/+CN/+CP/+NP/+CNP), Comp = compartment (litter or soil). Significant terms are displayed in boldface: *P < 0.05, **P < 0.01, ***P < 0.001.

Regardless of fertilization, microbial biomass C, N, and P (Cmic, Nmic and Pmic) were three- to sevenfold higher in the litter than in the soil (Table 1). Fertilization treatments significantly affected microbial biomass C, N, and P (Table 1) and stoichiometry in both, the litter and the soil (Table 1, Table S3). These fertilization effects were mainly driven by +P additions, with additional positive interactions with +C and +N in litter, but not in soil (Table S3). Scaling relationships showed that Pmic increased more rapidly (slope >1) than Nmic or Cmic in the soil microbial biomass compared to that in the litter (Fig. 2), especially in response to P additions (Fig. S1). This translated into lower N:Pmic (on average −16%) and C:Pmic ratios (−27%) in soil microbial communities compared with those in the litter (Fig. 3). Microbial stoichiometry varied from 88:9:1 to 124:12:1 in the litter compared to 59:7:1 to 105:10:1 in the soil. Coefficients of variation of C:N:Pmic were similar between litter and soil (Table 2).

**Table 1:** Litter and soil microbial biomass C, N and P and their ratios (mean ± SD, *n* = 5 per treatment) in response to fertilization treatments.

	Without phosphorus				With phosphorus				C.V.
	Ctrl	+C	+CN	+N	+P	+CP	+NP	+CNP	
*Litter Biomass and Ratios*									
Cmic (µg g^−1^)	1073.4 ± 360.2^ab^	1372.4 ± 530^abc^	828.5 ± 295.3^a^	1299.3 ± 879.5^ab^	1768.2 ± 613^bc^	1863.9 ± 584.6^bc^	2220.9 ± 953.8^c^	1398.4 ± 475.1^abc^	30.5
Nmic (µg g^−1^)	123.8 ± 68.9^ab^	131.8 ± 22.4^ab^	65.6 ± 24.3^a^	172.9 ± 40.3^bc^	226.3 ± 50^bc^	248.3 ± 115.9^c^	214.2 ± 45.6^bc^	165.2 ± 43.9^abc^	36.0
Pmic (µg g^−1^)	10.6 ± 6.9^ab^	11.1 ± 3.1^abc^	6.4 ± 1.7^a^	11.5 ± 2.6^abc^	20.1 ± 5.3^bcd^	22.3 ± 6.2 ^cd^	25.2 ± 12.4^abc^	15.8 ± 4.5^bcd^	42.8
C:Nmic	10.4 ± 5.2^ab^	10.2 ± 2.8^ab^	13.5 ± 4^b^	8.6 ± 2.2^a^	8.2 ± 2.3^ab^	7.4 ± 4.3^ab^	10.6 ± 4.2^ab^	8.0 ± 2.9^ab^	20.7
N:Pmic	12.1 ± 1.9^ab^	12.3 ± 2.4^ab^	10.1 ± 2.3^a^	10.5 ± 1.2^b^	10.8 ± 2.7^ab^	15.2 ± 3.3^ab^	9.6 ± 3.2^a^	11.6 ± 2.8^ab^	15.3
C:Pmic	121.5 ± 51.3^a^	121 ± 14.3^a^	128.5 ± 27.9^a^	88.5 ± 16.1^a^	86.5 ± 27.6^a^	105.3 ± 51.2^a^	93.9 ± 34.9^a^	87 ± 13.4^a^	16.8
*Soil Biomass and Ratios*									
Cmic (µg g^−1^)	245.8 ± 157.2^ab^	215.2 ± 101.2^a^	179.1 ± 55.9^a^	227.8 ± 73.6^ab^	255 ± 49.4^ab^	403.7 ± 74.9^b^	296.6 ± 30.6^ab^	286.8 ± 133.7^ab^	25.8
Nmic (µg g^−1^)	28.6 ± 7.6^abc^	19.9 ± 5.8^a^	21.9 ± 9^ab^	31.1 ± 8.9^abc^	37.1 ± 13.8^abc^	51.5 ± 23.5^c^	46.2 ± 18.4^bc^	38.6 ± 9.7^abc^	32.3
Pmic (µg g^−1^)	2.9 ± 1.8^a^	2.0 ± 0.7^a^	2.0 ± 0.6^a^	2.7 ± 0.6^a^	4.7 ± 1.0^ab^	6.9 ± 2.3^b^	5.9 ± 2^b^	4.0 ± 1.9^ab^	46.7
C:Nmic	8.4 ± 4.2^a^	11.3 ± 4.3^a^	8.6 ± 2.1^a^	7.3 ± 2.1^a^	8.7 ± 2.7^a^	7.8 ± 3.2^a^	7.3 ± 3^a^	7.8 ± 3.9^a^	15.3
N:Pmic	11.7 ± 5.1^a^	10 ± 3.1^a^	11.1 ± 2.2^a^	10.3 ± 2.1^a^	7.5 ± 1.7^a^	10.9 ± 2.8^a^	7.7 ± 0.6^a^	8 ± 2.3^a^	17.4
C:Pmic	82.5 ± 3.6^abc^	102.8 ± 20.6^c^	92.3 ± 17.9^bc^	72.2 ± 5.9^abc^	64.8 ± 27^a^	80.3 ± 26.2^ab^	54.6 ± 18.4^abc^	54.9 ± 3^abc^	22.9

Different letters indicate significant differences among treatments.

**Table 2:** Comparison of coefficient of variations between litter and soil for C:Nmic, N:Pmic, and C:Pmic ratios.

CV	Test name	Test statistic	*P-value*
C:Nmic	Asymptotic test	0.03	0.87
	M-SLRT	0.02	0.90
N:Pmic	Asymptotic test	1.56	0.21
	M-SLRT	1.52	0.22
C:Pmic	Asymptotic test	0.19	0.67
	M-SLRT	0.19	0.67

We used two different tests to assess whether coefficients of variation (CV) differ between litter and soil: the ‘asymptotic test’ for CV equality and the ‘modified signed-likelihood ratio test’ (M-SLRT) (R package ‘cvequality’). Non-significant *p-values* indicate no difference in CVs between litter and soil.

### Stoichiometric plasticity of microbial communities

**Figure 4:**
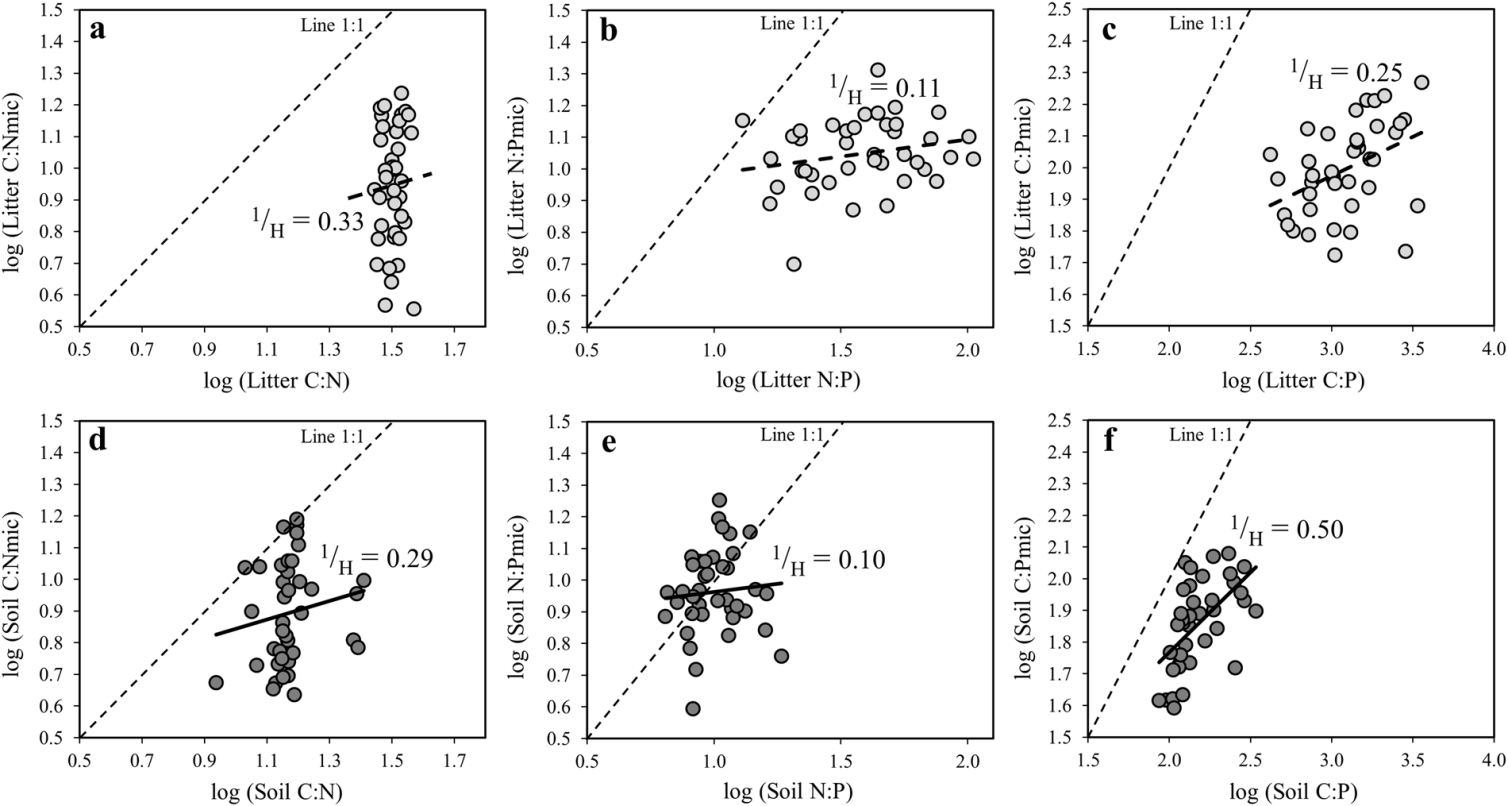
Relationships between log_10_-transformed stoichiometries of microbial biomass and their substrates for C:N (**a**), NP (**b**), and litter C:P (**c**) ratios (light grey circles, dashed line, *n* = 40) and C:N (**d**), N:P (**e**), and soil C:P (**f**) ratios (dark grey circles, full line, *n* = 39). 0 < ^1^/_H_ < 0.25-‘homeostatic’; 0.25 < ^1^/_H_ < 0.5-‘weakly homeostatic’; 0.5 < ^1^/_H_ < 0.75-‘weakly plastic’; ^1^/_H_ > 0.75-‘plastic’.

Scaling relationships between the stoichiometry of microbial biomass and that of their substrates indicated weakly homeostatic (C:N, ^1^/_H_ = 0.33 in litter and 0.29 in soil) to homeostatic (N:P, ^1^/_H_ = 0.11 in litter and 0.10 in soil) regulation of microbial stoichiometry (Fig. 4). However, while C:Pmic in litter communities was weakly homeostatic (^1^/_H_ = 0.25), it was weakly plastic in soil communities (^1^/_H_ = 0.50). This response was mostly driven by P fertilization leading to a clear separation between soils with or without P fertilization (Fig. S2).

## Discussion

Our aim was to evaluate the impact of distinct substrate stoichiometry affected by additional fertilization on the C:N:P stoichiometry of microbial communities in the leaf litter layer and in the underlying soil. Because leaf litter presented wider C:N:P ratios (1048:33:1 on average) than the underlying soil (151:10:1), we expected that the stoichiometry of microbial biomass would reflect these differences to some extent. In support of our first hypothesis, litter communities presented wider C:N:Pmic ratios (96:11:1 on average) compared to soil communities (68:9:1). Wider stoichiometric ratios may be generated by adjustments in growth and resource allocation at the organismal level or by shifts in the structure of microbial communities^12,13^. In a previous experiment with litter and soil from the same study site, there were indeed much higher fungi to bacteria ratios in litter (ranging from 0.86 to 2.90^14^) than in soil (ranging from 0.21 to 0.23^6^). Differences in carbon-use efficiency between bacteria and fungi^15^ coupled with lower nutrient demand of fungi compared to bacteria^16^ may lead to different substrate use and higher relative abundance of fungi on substrates with wide C:N:P ratios. The relative availability of nutrients in mineral *versus* organic form and the changing quality of decomposing litter over time may additionally control the relative abundance of fungi and bacteria and community stoichiometry. For a more detailed understanding of how different microbial groups cope with stoichiometric imbalance of their resources and the dynamic changes in growth adjustments and community structure that may operate simultaneously, microbial community composition and abundance would need to be assessed with high taxonomic resolution.

In line with an expected strong P limitation of heterotrophs in our study system^17^, microbial biomasses of both litter and soil communities were strongly stimulated by P additions. Yet, we did not observe that C:N:Pmic in litter varied more (from 88:9:1 to 124:12:1) than that in the soil (from 59:7:1 to 105:10:1). Contrary to our second hypothesis, these results highlight that litter microbial communities are not stoichiometrically more plastic than those in the soil, but they just vary at overall higher ratios. Interestingly, we observed that C:nutrient ratios were less constrained than N:P ratios in both litter and soil communities. This suggests that stoichiometric plasticity arises mostly because of a decoupling between C and nutrients, which might reflect higher variation in microbial biomass C potentially due to storage of C compounds. On the other hand, the relative amounts of P and N may vary less because P in RNA and N in proteins are much tighter linked metabolically^18^.

The relative plasticity in the stoichiometry of the microbial biomass combined with the critical role of P are important for the understanding of the regulation of the C and nutrient cycles and they should be considered in theoretical modeling. Our findings do not support the simplifying premise of strict homeostasis in heterotrophs, and changes in resource availability and stoichiometry may have important feedbacks on nutrient dynamics. Also, our results highlight that the elemental ratios were wider in microbial communities of the litter layer than those in soil microbial communities. This difference strongly suggests that microbial communities in the litter layer should be considered apart of those in the underlying soil when studying microbial limitations along the litter-soil continuum. Nevertheless, the difference between substrate stoichiometry and microbial biomass stoichiometry is much larger in litter communities compared to soil communities. This could indicate a decrease in the carbon-use efficiency^19^ coupled to an increase in nitrogen-use efficiency^3^ in litter microbes compared to soil microbes. Alternatively and as shown before, the stoichiometry of bulk litter may not well represent the actual resources used by litter microbes, which may rather respond to litter leachate stoichiometry^14^. Further studies investigating the composition of microbial communities in relation to the element-use efficiency will be necessary to disentangle the effects of changes in the physiology of microbes at the organismal level from those related to the shifts in the microbial community structure at the community level.

## Methods

### Field site

The study site is located in an undisturbed Amazonian rainforest at the Paracou experimental station in French Guiana (5°18′N, 52°53′W). The mean annual air temperature is 25.7 °C and the mean total annual rainfall is 3041 mm (1971–2001). The forest is composed of about 140 species ha^−1^ with a mean density of 620 individual trees ha^−1^ (individuals of diameter >0.1 m at breast height) and an average tree height of 35 m^20^. Soils in the study area are classified as nutrient-poor acrisol (FAO-ISRIC-ISSS) developed over a Precambrian metamorphic formation called the Bonidoro series. The soil texture is characterized by 20% clay, 6% silt and 74% sand, and a pH (water extract) of 4.4 in the top 0.08 m. Average soil C:N:P before fertilization was 223:15:1 with a total C of 22.4 g kg^−1^, a total N of 1.5 g kg^−1^ and a total P of 0.10 g kg^−1^ soil.

### Fertilization experiment

A full factorial fertilization experiment was set up with C, N and P addition in all possible combinations (control without any fertilization/+C/+N/+P/+CN/+CP/+NP/+CNP). These eight treatments were applied to 5.5 × 5.5 m plots within five blocks distributed in a 2.5-ha zone. We added C as cellulose (commercial substrate Waterspare, Celliob industry, France) corresponding to 0.5 times the annual natural C-input to the soil via leaf litter fall (*i.e*. 1405 kg C ha^−1^ year^−1^), N as urea corresponding to twice the annual natural N input from leaf litter (*i.e*. 130 kg N ha^−1^ year^−1^), and P as KH_2_PO_4_ corresponding to 50 fold the annual natural P input via leaf litter fall (*i.e*. 69 kg P ha^−1^ year^−1^, see Barantal *et al*.^21^ for further details). The added doses of nutrients were chosen so as to alleviate limitations of microbial communities and are comparable to those used in other fertilization experiments in tropical rain forests^22–24^. Fertilizers were applied twice a year from April 2009 to 2011. Cellulose was chosen as C fertilizer rather than glucose or any other sugar because it represents the naturally available C sources much better, and also to avoid an artificially strong and immediate response of opportunistic microorganisms. Organic rather than mineral sources were used for N fertilization to avoid rapid leaching (particularly of nitrate) out of the experimental plots exposed to regular and heavy rainfall. Pre-treatment concentrations of C, N, and P in litter and soil varied among experimental blocks^25^, which increased the range of substrate stoichiometry along which we could test our hypotheses. Despite these pre-treatment differences, the fertilization treatments had the same consistent effects on changes in resource stoichiometry across blocks, and the P fertilization effect was much stronger than the pre-treatment differences (Fig. 1).

### Litter and soil collection

We used the natural litter produced by trees from each plot to construct litterbags in order to use the original site-specific litter mixture for the decomposition experiment. After collection, leaf litter from the first year of fertilization (May 2009-April 2010) was pooled for each plot across sampling dates. Leaves with obvious signs of damage (*e.g*. herbivory, galls, fungal attacks) and green leaves were excluded (typically <15% of total collected leaves). Litter was air-dried, weighed (40.0 ± 0.1 g per litterbag) and enclosed in two plastic mesh bags (coarse-mesh size of 8 mm) for each plot individually. Two 0.15 × 0.15 m large litterbags were randomly placed directly on the soil surface (natural litter was removed prior to litterbag placement), fixed on the forest floor with wire and exposed in each of the 45 plots for a total of four months from October 2010 (just before the fourth fertilization event) to February 2011^17^. After 107 days of exposure in the field, the litterbags were retrieved and the underlying soil underneath each litterbag was collected as follows: the soil was sampled in the center of the litterbag using a stainless steel cylinder (diameter of 0.05 m) to a depth of 0.08 m. We chose this duration of field exposure for two reasons: i) the litter is in its initial (linear) stage of decomposition with about 20–30% of mass loss after roughly 4 months^21,26^; and ii) to harvest sufficient litter material left for the different analyses. Because of the quantity necessary for different analyses (about 20 g) and the rapid litter mass loss in some treatments, we pooled the two litterbags per plots and used the same procedure for the soil samples corresponding to a total of 90 litter and 90 soil samples (9 treatments × 5 blocks × 2 compartments). All sampling was done from 6^th^ to 10^th^ February 2011 during the wet season. In the laboratory, litter from the litterbags was weighed for total fresh mass and stored refrigerated for biomass analyses while an aliquot (5 g fresh weight) was dried at 65 °C to determine litter mass loss and used for chemical analyses. Soil samples were passed through a 2 mm sieve to remove roots and stones, homogenized and stored refrigerated for biomass analyses, while an aliquot (5 g fresh weight) was air-dried fore chemical analyses.

### Microbial biomass

Carbon, N and P in the microbial biomasses (Cmic, Nmic and Pmic) were measured using the chloroform fumigation extraction method according to Brookes *et al*.^27^. Upon harvest, four approximately 5 g (fresh weight)-aliquotes for each litter sample or 10 g (fresh weight) for soil samples were taken. Two aliquotes were fumigated for 24 h with chloroform and extracted with 3:1 (v:w) 0.5 M of K_2_SO_4_ for total C and N or 0.5 M of NaHCO_3_ for total P. The two other subsamples were directly extracted according to the same protocol for non-microbial biomass C, N and P contents. The extracts were then filtered with pre-soaked Whatman 42 paper. Total P contents were determined colorimetrically using the molybdenum blue method^28^, and total C and N contents were determined by combustion at the Laboratoire d’Analyse des Sols (INRA, Arras-France). The microbial biomass element contents were calculated from the difference between fumigated and non-fumigated samples and adjusted using empirically-derived conversion factors (0.45, 0.45 and 0.40 for C, N and P, respectively Jenkinson *et al*.^29^), and expressed per unit of litter or soil dry mass.

### Litter and soil chemical analyses

All chemical analyses were performed after the final harvest on litter and soil subsamples that were ground to obtain a uniform particle size of about 1 mm (Cyclotech Sample Mill, Tecator, Höganäs, Sweden). Total C and N concentrations were measured using 5 mg DW litter or 80 mg DW soil with a CHN elemental analyzer (Flash EA1112 Series, ThermoFinnigan, Milan, Italy). For P measurements, 2 ml of H_2_SO_4_ 36 N and 3 ml of H_2_O_2_ were added to a 25 mg of litter or 150 mg of soil, and heated at 360 °C for 4 h. After this mineralization step, P concentration was measured colorimetrically with an autoanalyzer (Evolution II, Alliance Instruments, Frépillon, France) using the molybdenum blue method^28^.

### Statistical analysis

We evaluated the effects of fertilizer addition on substrate stoichiometry by comparing litter layer and soil stoichiometry before the fertilizer treatments started^25^ with those after two years of fertilization (at the end of our litter decomposition experiment) for each plot and corrected by the variation over time in control plots. Substrate C, N and P as well as Cmic, Nmic and Pmic data were analyzed using linear mixed models, with fertilization treatments and substrate type (litter *vs* soil) as fixed factors, and blocks as random factors. The same model using a factorial design was used to compare all plots receiving +C, +N or +P with all plots that were not fertilized with the respective resource. The relationships among Cmic, Nmic and Pmic were examined using standardized major axis; we then evaluated the heterogeneity in slopes between litter and soil C:N:Pmic. Coefficient of variation representing the range of variation in microbial C:Nmic, N:Pmic and C:Pmic ratios were compared using asymptotic^30^ and modified signed-likelihood ratio^31^ tests. Finally, we calculated the degree of stoichiometric homeostasis for litter and soil communities using the ^1^/_H_ coefficient^32,33^ that correspond to the slope of the regression between log(resource) and log(biomass), and we applied the classification suggested by Persson *et al*.^34^: 0 < ^1^/_H_ < 0.25-‘homeostatic’; 0.25 < ^1^/_H_ < 0.5-‘weakly homeostatic’; 0.5 < ^1^/_H_ < 0.75-‘weakly plastic’; ^1^/_H_ > 0.75-‘plastic’.

### Data accessibility

https://knb.ecoinformatics.org/#view/knb.1314.2

## Electronic supplementary material

Supplementary Information
