# Supplementary material for: Stoichiometric plasticity of microbial communities is similar between litter and soil in a tropical rainforest
Source: Sci Rep. 2017 Oct 2;7:12498. doi: 10.1038/s41598-017-12609-8 (PMC5624877; doi:10.1038/s41598-017-12609-8)

Supplementary Information for:

Stoichiometric plasticity of microbial communities is similar between litter and soil in a tropical rainforest

Author names. Nicolas Fanin[1*], Nathalie Fromin[2+], Sandra Barantal[3], Stephan Hättenschwiler[2]

Affiliations. [1]Interaction Soil Plant Atmosphere (ISPA), UMR 1391, INRA - Bordeaux Sciences Agro, 71 avenue Edouard Bourlaux, 33882 Villenave-d'Ornon cedex, France

[2]Centre of Evolutionary and Functional Ecology (CEFE), UMR 5175, CNRS - Université de Montpellier - Université Paul-Valéry Montpellier - EPHE, 1919 route de Mende, 34293 Montpellier, France

[3]School of Biological Sciences, Royal Holloway, University of London, Egham, United Kingdom

[*]nicolas.fanin@inra.fr

[+]current address: PROMES-CNRS, 7 rue du Four Solaire, F-66120 Odeillo, France

Summary:

Table S1 | Concentrations and ratios of C, N and P in litter and soil in response to fertilization treatments (mean ± SD, *n* = 5 per treatment)…………………………………………………………..………………3

Table S2 | Results from full factorial mixed linear models to test for the effects of fertilization (addition or not of either one of C, N and P) and their interactions on substrate stoichiometry……………………………4

Table S3 | Results from full factorial mixed linear models to test for the effects of fertilization (addition or not of either one of C, N and P) and their interactions on microbial stoichiometry.……….…………………5

Figure S1 | Relationships between C, N and P in the microbial biomass (mean ± SD) for litter and soil…..…6

Figure S2 | Relationships between microbial biomass and substrate stoichiometry (log_{10}-transformed) in litter and soil………………….…...…...…………………………………………………………………………7

Table S1 | Concentrations and ratios of C, N and P in litter and soil in response to fertilization treatments (mean ± SD, *n* = 5 per treatment). Different letters indicate significant differences among treatments.

	Without phosphorus				With phosphorus			
	Ctrl	+C	+CN	+N	+P	+CP	+NP	+CNP
Litter Elements and Ratios								
C (g kg^{-1})	48.9±1.1^{a}	45.9±2.7^{a}	45.8±3.5^{a}	48.0±1.1^{a}	47.9±4.1^{a}	47.4±1.2^{a}	49.0±0.6^{a}	46.6±2.5^{a}
N (g kg^{-1})	1.56±0.12^{a}	1.38±0.11^{a}	1.43±0.06^{a}	1.49±0.15^{a}	1.56±0.16^{a}	1.53±0.09^{a}	1.50±0.09^{a}	1.48±0.04^{a}
P (g kg^{-1})	0.029±0.009^{a}	0.024±0.006^{a}	0.024±0.004^{a}	0.024±0.009^{a}	0.046±0.003^{ab}	0.081±0.028^{c}	0.060±0.010^{bc}	0.069±0.012^{bc}
C:N	31.5±2.4^{a}	33.3±2.3^{a}	32.0±2.5^{a}	32.4±3.0^{a}	30.7±1.9^{a}	30.9±2.1^{a}	32.6±2.1^{a}	31.5±2.3^{a}
N:P	59.5±26.3^{bc}	59.0±17.4^{bc}	58.9±10.7^{bc}	67.5±24.0^{c}	33.4±2.5^{ab}	22.1±11.9^{a}	25.6±5.1^{a}	21.5±2.8^{a}
C:P	1904.6±957.8^{bcd}	1979.5±628.8^{cd}	1900.8±447.8^{bcd}	2218.9±894.3^{d}	1024.4±18.8^{abc}	693.4±412.9^{a}	835.0±153.4^{ab}	684.8±123.5^{a}
Soil Elements and Ratios								
C (g kg^{-1})	2.39±0.99^{a}	2.30±0.62^{a}	2.14±0.44^{a}	2.27±0.54^{a}	1.68±0.41^{a}	1.75±0.26^{a}	1.70±0.34^{a}	1.91±0.36^{a}
N (g kg^{-1})	0.15±0.06^{a}	0.13±0.04^{a}	0.12±0.02^{a}	0.12±0.04^{a}	0.11±0.02^{a}	0.13±0.02^{a}	0.13±0.04^{a}	0.14±0.03^{a}
P (g kg^{-1})	0.012±0.002^{abc}	0.010±0.002^{a}	0.011±0.003^{abc}	0.010±0.002^{ab}	0.014±0.003^{bc}	0.016±0.002^{c}	0.015±0.003^{c}	0.015±0.003^{c}
C:N	15.2±1.15^{a}	17.1±4.08^{a}	18.3±5.94^{a}	18.7±5.23^{a}	14.3±0.56^{a}	12.9±1.31^{a}	13.5±3.42^{a}	13.5±1.63^{a}
N:P	12.4±3.37^{bc}	13.6±1.74^{c}	10.5±1.84^{abc}	11.5±2.55^{abc}	7.98±0.63^{a}	8.60±1.68^{ab}	8.74±2.19^{ab}	9.29±1.30^{ab}
C:P	190.9±58.4^{abc}	230.8±41.9^{c}	201.6±100.2^{bc}	211.4±45.1^{c}	114.4±12.4^{a}	110.6±19.5^{a}	112.5±7.0^{a}	124.1±5.8^{ab}

Table S2 | Results from full factorial mixed linear models to test for the effects of fertilization (addition or not of either one of C, N and P) and their interactions on substrate stoichiometry. Significant effects are displayed in boldface (**$P < 0.01$, ***$P < 0.001$). There were no significant interactions.

Source of variation	d.f.	F-value	*P*-value		d.f.	F-value	*P*-value
(a) *Litter stoichiometry*				(b) *Soil stoichiometry*			
C:N				C:N			
+C	32	0.04	0.84	+C	32	0.00	1.00
+N	32	0.54	0.47	+N	32	1.46	0.24
+P	32	1.61	0.21	+P	32	12.48	**0.001****
N:P				N:P			
+C	32	1.73	0.20	+C	32	0.38	0.54
+N	32	0.01	0.97	+N	32	1.18	0.29
+P	32	59.73	**<0.001*****	+P	32	29.42	**<0.001*****
C:P				C:P			
+C	32	1.14	0.2931	+C	32	0.47	0.50
+N	32	0.01	0.9559	+N	32	0.01	0.95
+P	32	49.38	**<0.001*****	+P	32	46.05	**<0.001*****

Table S3 | Results from full factorial mixed linear models to test for the effects of fertilization (addition or not of either one of C, N and P) and their interactions on microbial stoichiometry. Only significant interaction terms are included in the final models. Significant effects are displayed in boldface (**P* < 0.05, ***P* < 0.01, ****P* < 0.001).

Source of variation	d.f.	F-value	*P*-value		d.f.	F-value	*P*-value
(a) *Litter microbial stoichiometry*				(b) *Soil microbial stoichiometry*			
C:Nmic				C:Nmic			
+C	30	1.23	0.28	+C	31	1.27	0.27
+N	30	0.77	0.39	+N	31	1.79	0.19
+P	30	2.85	0.10	+P	31	1.52	0.23
+C × +P	30	4.58	**0.041***				
+C × +N × +P	30	5.36	**0.028***				
N:Pmic				N:Pmic			
+C	31	2.13	0.16	+C	31	0.10	0.76
+N	31	0.22	0.64	+N	31	0.66	0.42
+P	31	4.89	**0.035***	+P	31	9.34399	**0.005****
+C × +N × +P	31	4.90	**0.035***				
C:Pmic				C:Pmic			
+C	32	0.27	0.60	+C	31	8.34	**0.007****
+N	32	0.00	0.99	+N	31	0.15	0.70
+P	32	14.08	**<.001*****	+P	31	26.74	**<.001*****

Figure S1 | Relationships between C, N and P in the microbial biomass (mean ± SD) for litter (a, b, c) and soil (d, e, f). The eight fertilization treatments (n = 5 per treatment) were separated graphically into two sub-groups: those that received P (red diamonds; +P, +CP, +NP, +CNP) and those that did not (white circles; ctrl, +C, +N, +CN).

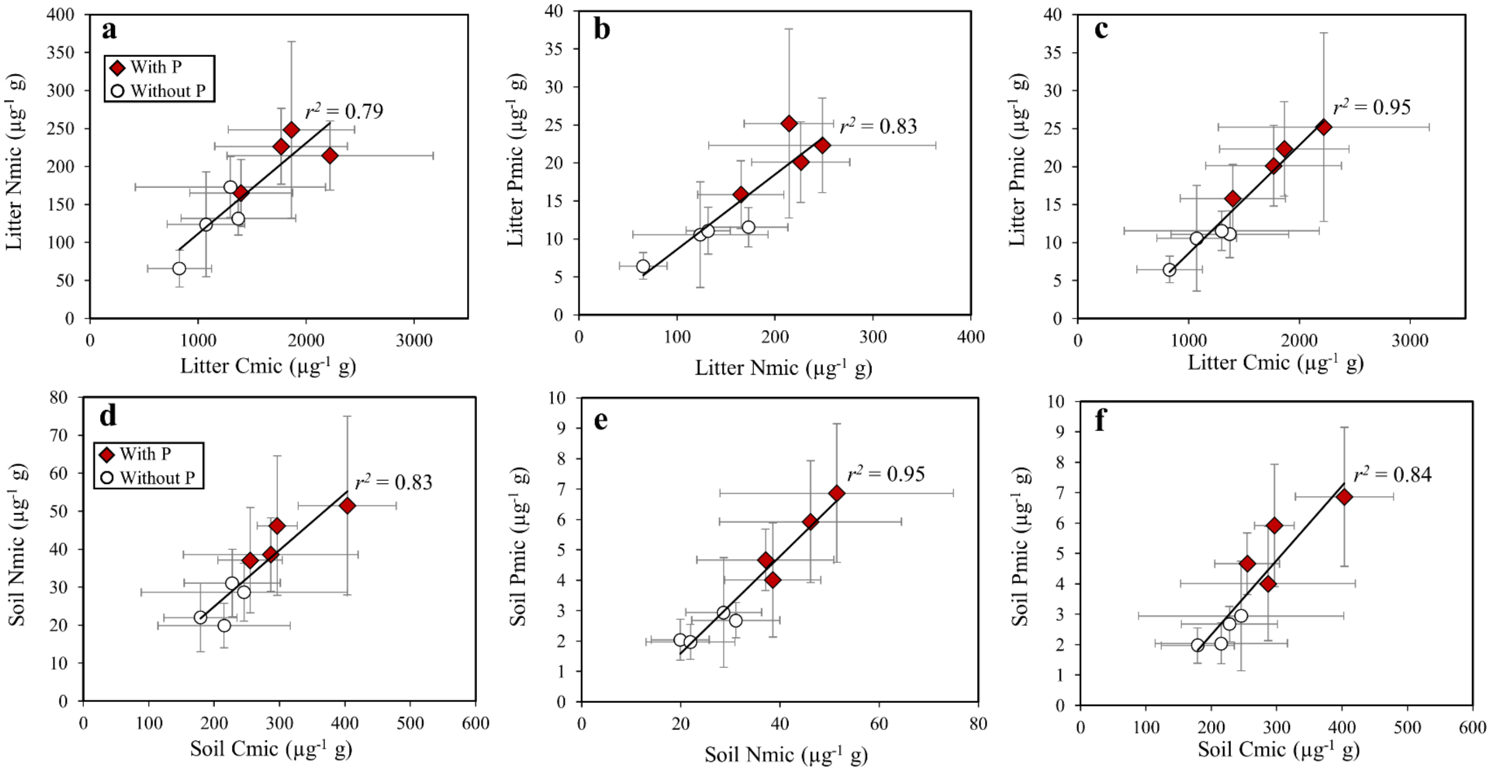

Figure S2 | Relationships between microbial biomass and substrate stoichiometry (log_{10}-transformed) in litter (a, b, c) and soil (d, e, f). The eight fertilization treatments (*n* = 5 per treatment) were separated graphically into two sub-groups: those that received P (red diamonds; +P, +CP, +NP, +CNP) and those that did not (white circles; ctrl, +C, +N, +CN).

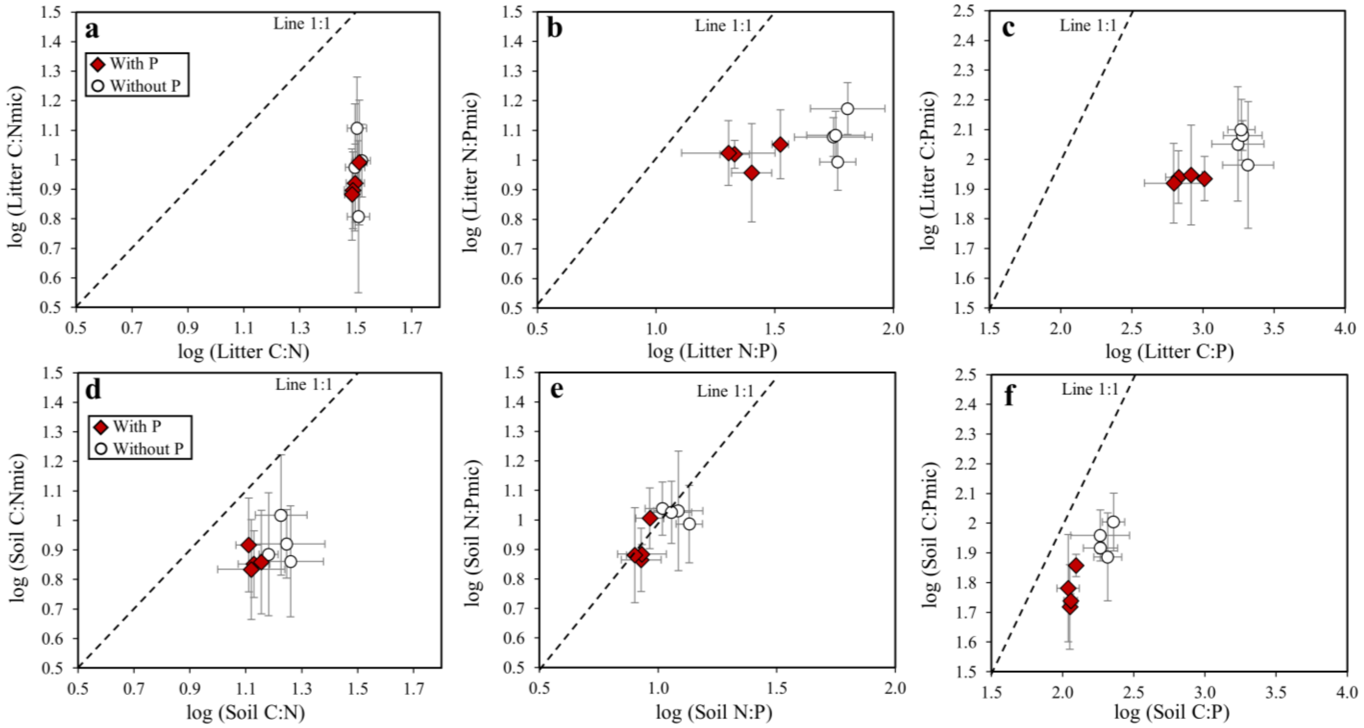

Supplement: Supplementary file 1 — Supplementary Information [file 41598_2017_12609_MOESM1_ESM.pdf]
